# A Scoping Review of Synthetic Data Generation by Language Models in Biomedical Research and Application: Data Utility and Quality Perspectives

**DOI:** 10.1007/s41666-026-00229-9

**Published:** 2026-02-02

**Authors:** Hanshu Rao, Weisi Liu, Haohan Wang, I-Chan Huang, Zhe He, Xiaolei Huang

**Affiliations:** 1 Department of Computer Science, University of Memphis, Memphis, 38152 TN United States; 2 School of Information Sciences, University of Illinois Urbana-Champaign, Champaign, 61820 IL United States; 3 Epidemiology and Cancer Control, St Jude Children’s Research Hospital, Memphis, 38105 TN United States; 4 School of Information, Florida State University, Tallahassee, 32306 FL United States

**Keywords:** Synthetic data generation, Language model, Biomedical informatics, Data quality

## Abstract

Synthetic data generation using large language models (LLMs) demonstrates substantial promise in addressing biomedical data challenges and shows increasing adoption in biomedical research. This study systematically reviews recent advances in synthetic data generation for biomedical applications and clinical research, focusing on how LLMs address data scarcity, utility, and quality issues with different modalities. We conducted a scoping review following PRISMA-ScR guidelines and searched literature published between 2020 and 2025 through PubMed, ACM, Web of Science, and Google Scholar. A total of 59 studies were included based on relevance to synthetic data generation in biomedical contexts. Among the reviewed studies, the predominant data modalities were unstructured texts (78.0%), tabular data (13.6%), and multimodal sources (8.4%). Common generation methods included LLM prompting (74.6%), fine-tuning (20.3%), and specialized models (5.1%). Evaluations were heterogeneous: intrinsic metrics (27.1%), human-in-the-loop assessments (44.1%), and LLM-based evaluations (13.6%). However, limitations and key barriers persist in data modalities, domain utility, resource and model accessibility, and standardized evaluation protocols. Future efforts may focus on developing standardized, transparent evaluation frameworks and expanding accessibility to support effective applications in biomedical research.

**Supplementary Information:**

The online version contains supplementary material available at 10.1007/s41666-026-00229-9.

## Introduction

Data is the key to train reliable AI models for broad biomedical and clinical applications, such as medical diagnosis [1, 2], therapeutic treatments [3], and drug discovery [4]. However, obtaining massive, privacy-preserving, and high-quality data faces critical challenges, such as data availability, noisy and missing data, and legal regulations. Increasing biomedical studies are turning attention to synthetic data generation, a process of creating artificial datasets that accurately replicate the statistical and structural properties of real-world data. Nonetheless, creating high-quality synthetic data remains challenging due to the inherent heterogeneity, complexity, and variability characteristic of biomedical data. Large language models (LLMs) may offer a promising solution by enhancing the availability and utility of synthetic datasets in critical applications, such as clinical note analysis for stroke thrombolysis contraindication identification [5], mental health diagnosis through automated interview assessment [6], and radiology report generation for fracture misdiagnosis detection [7].

**Fig. 1:**
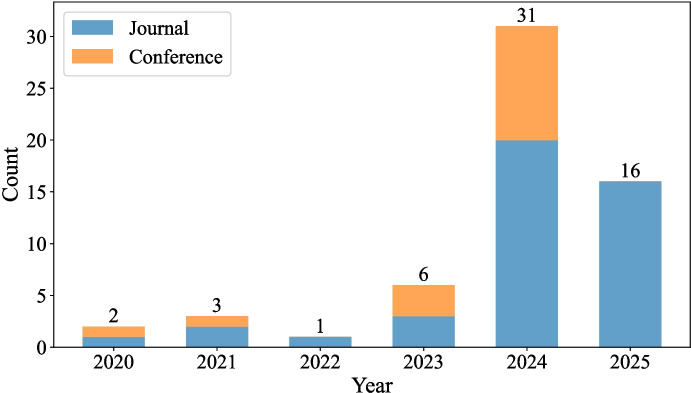
Publications between Jan 01, 2020 and April 05, 2025. Orange and blue colors refer to peer-reviewed conference and journal publications, respectively. We can observe a surging increase on the biomedical synthetic data related studies

Synthetic data generation in biomedical and clinical fields has achieved significant advances by LLMs (e.g., GPT-4 [8] and Llama 3 [9]) in recent years, as shown in Figs. 1 and 4. For example, LLMs have been applied successfully to obtain clinical narratives and simulate real patient records of mental health for diagnosis and behavior analysis [10], such as predicting suicide [11] and depression patterns [12]. The strong generation capability not only relieves data privacy concerns [13] to facilitate model developments but also may promote data quality [14] to support clinical decision-making. For example, synthetic corpora have augmented classification models for cardiovascular and Alzheimer’s disease diagnosis [15]; and a study develops a multi-agent dialogue generator (NoteChat) that creates synthetic patient-physician conversations to improve clinical documentation [16]. Nevertheless, questions and uncertainties remain regarding the best practices, validation approaches, and specific application areas for those LLM-generated synthetic data.

The goal of this scoping review is to comprehensively summarize and assess recent research publications on the biomedical and clinical utilities leveraging LLMs for synthetic data generation and its quality evaluation. While multiple reviewing studies [17–19] have covered LLM-generated synthetic data for general domains, studies on biomedical and clinical domains remain underexplored. For example, a close study [19] examines recent developments of generation algorithms and models on news and social media domains instead of biomedical fields. Several recent review studies [20–23] have examined the topic in health and clinical fields, however, their focus on methodologies and models often overlooks fundamental aspects of synthetic data itself, such as types, utility, accessibility, and data quality –– gaps that this study aims to address. Specifically, through this study, we will identify and present the current state-of-the-art models, technical methods, evaluations or assessments of synthetic data quality, and gaps and opportunities for future research. We seek to answer a concrete yet unsolved question: *what biomedical and clinical applications can be effectively addressed using LLM-generated synthetic data, and how?*

## Results

Our search and initial review resulted in 132 articles and finalized with 59 articles for this scoping review. XH, HW, and HR reviewed full texts of the articles and removed 73 articles due to ineligible article types ($n=2$), no large language model ($n=7$), unrelated to biomedical data generation ($n=18$), and not peer-reviewed and published in a journal or conference proceedings ($n=46$). Inter-rater reliability, assessed using Cohen’s $\kappa$ [24], indicated almost perfect agreement for both screening phases ($\kappa =0.96$ for title/abstract screening, $n=307$; $\kappa =0.95$ for full-text screening, $n=132$). Fifty-nine articles met our criteria and were included in our subsequent analyses. In terms of publication venues and formats, the included literature consists of 43 journal articles (72.9%) and 16 peer-reviewed conference papers (27.1%), leaving 59 studies to be included in this scoping review. Figure 1 shows that the study number of biomedical synthetic data generation climbs steadily over the years. Particularly, the substantial growth after 2022 reflects a particularly evolving trend of synthetic biomedical data generation via LLM, an emerging generation tool in recent years.

We present an overview of recent progress in synthetic biomedical data generation and application from three perspectives, aiming to provide readers with an insightful understanding of current practices and inform future research directions:

1. Synthetic data types and medical applications: Research has explored broad synthetic data types of clinical text, tabular data, and multimodal information (e.g., images, audio, and sensor signals). These data types cover a range of applications, including EHR analysis, medical imaging, telemedicine, mental health, and clinical trial matching, addressing challenges related to data scarcity and privacy restrictions.
2. Synthetic data generation methodology: Evolving methodologies encompass prompting-based generation, knowledge infusion, and multi-agent and multimodal approaches. These advancements enhance the semantic consistency, efficiency, and scalability of synthetic biomedical data generation.
3. Quality assessments and evaluation metrics: Ensuring data quality and utility remains a priority. Current studies employ evaluation strategies targeting fidelity, utility, and privacy protection. Such strategies integrate statistical metrics, human-in-the-loop review, automated model assessments, and privacy testing to establish a robust framework for verifying the reliability and compliance of synthetic data.

**Table 1:** Summary of data types, generation methods, accessibility, purposes and medical applications in the collected studies. Languages include English (EN), Dutch (NL), French (FR), Chinese (ZH), and Arabic (AR). Generation methods comprise fine-tuning (SFT: supervised, DAFT: domain-adaptive, TAFT: task-adaptive, IT: instruction tuning, None) and prompting (ZS: zero-shot, FS: few-shot, INST: instruction-driven, CoT: reasoning-augmented, RAG: knowledge-augmented). Synthetic data purpose includes Training (used independently), Supplement (used with real data for joint training), and Privacy (used for privacy preservation such as sharing or de-identification). Data accessibility is classified as Yes (publicly available), Request (available upon request), and No (not publicly available or not specified). Medical applications cover question answering (QA), information extraction (IE), and social determinants of health (SDoH)

Study	Modality	Lang.	Year	Generative model	Approach		Accessible	Purpose	Medical application
					Fine-tuning	Prompting			
[25]	Text	EN	2020	BART	TAFT	INST	No	Training	Biomedical QA
[26]	Text	EN	2020	Transformer	SFT	INST	No	Training	Phenotype inference
[27]	Text	EN	2021	GPT-2	TAFT	ZS	Yes	Training	Clinical IE
[28]	Text	NL	2021	GPT-2	DAFT	ZS	No	Training	De-identification
[29]	Text	EN	2021	GPT-3	None	FS, RAG	No	Training	Dialogue summarization
[30]	Text	EN	2022	GPT-2	TAFT	ZS	No	Training	Readmission prediction
[31]	Text	EN	2023	GPT-3	None	ZS	Yes	Training	General
[32]	Text	FR	2023	GPT-2, Bloom	DAFT	ZS	Yes	Training	Clinical IE
[33]	Text	EN	2023	GPT-2	TAFT	ZS	No	Training	Syndromic detection
[34]	Text	EN	2023	GPT-3.5	IT	INST	Yes	Training	Assisted suicide
[35]	Tabular	EN	2023	HALO	TAFT	INST	Yes	Training	General
[15]	Text	EN	2023	Llama, GPT-4	None	CoT	Request*	Training	Alzheimer’s disease
[36]	Text	EN	2024	ChatGPT API	None	INST	Yes	Training	Discharge summary
[6]	Text	EN	2024	GPT-4	None	INST, CoT	Request*	Training	PTSD diagnosis
[37]	Image	ZH, EN	2024	GPT-3.5	None	INST	No	Training	Medical imaging
[38]	Text	EN	2024	GPT-3.5, Llama-2	None	INST	Yes	Training	Acute renal failure
[13]	Text	EN	2024	GPT-3.5	None	INST	No	Privacy	Phenotype inference
[39]	Text	EN	2024	GPT-4	None	INST, CoT	No	Training	Cohort selection
[40]	Text	EN	2024	GPT-3.5	None	INST, RAG	No	Supplement	Cohort selection
[41]	Text	DE	2024	Zephyr, GPT-4	None	INST, RAG	No	Training	Emergency medicine
[42]	Text	EN	2024	Mistral	None	ZS, INST	Yes	Training	Psychological therapy
[43]	Text	EN	2024	GPT-4, Llama-2	IT	ZS, FS	Yes	Training	Medical QA
[44]	Text	EN	2024	T5	TAFT	RAG	Request	Training	Medical QA
[14]	Text	EN	2024	GPT-3.5	None	FS, RAG	Yes	Training	General
[45]	Text	EN	2024	GPT-3.5/4	None	FS, INST	Yes	Supplement	SDoH extraction
[46]	Tabular	EN	2024	ChatGPT	None	FS	No	Supplement	Medical education
[16]	Text	EN	2024	GPT-3.5	None	INST, CoT	Yes	Supplement	Dialogue generation
[47]	Text	EN	2024	GPT-3.5	None	ZS, INST	No	Training	SDoH extraction
[48]	Tabular	EN	2024	GPT-4	None	FS	No	Training	Gait rehabilitation
[49]	Text	EN	2024	GPT-3.5	None	FS, INST	Yes	Training	General
[50]	Audio	EN	2024	GPT-4	None	INST	Yes	Supplement	EMS assistance
[51]	Text	EN	2024	Vicuna	None	INST	Yes	Training	Clinical IE
[11]	Text	EN	2024	GPT-3.5, Flan-T5, Llama-2	None	ZS, FS	No	Supplement	Suicide prediction
[52]	Text	EN	2024	GPT-4	None	INST	No	Training	Cancer symptom extraction
[53]	Text	EN	2024	ChatGPT	None	INST	Yes	Training	Clinical summary
[54]	Text	EN	2024	GPT-3.5	None	INST, RAG	Yes	Training	Medical QA
[55]	Tabular	-	2024	GPT-4o	None	FS, CoT	No	Supplement	Nursing care
[56]	Text	EN	2024	GPT-4	None	INST, ZS	Yes	Training	Patient portal triage
[57]	Text	EN	2024	Vicuna v1.1, Orca	IT	INST	No	Training	Report generation
[58]	Text	AR	2024	ChatGPT	None	INST, RAG	Yes	Supplement	Health chatbot
[59]	Tabular	EN	2024	ChatGPT	None	FS, INST	No	Supplement	Clinical trial simulation
[12]	Text	EN	2024	Llama, Alpaca	None	ZS, INST	No	Supplement	Depression symptom
[60]	Text	EN	2024	GPT-3.5/4	None	ZS, INST	No	Supplement	Autism diagnosis
[5]	Text	EN	2025	ChatGPT, Llama, Gemini	None	INST	Request	Privacy	Stroke prediction
[61]	Audio	EN	2025	GPT-4	None	FS, CoT	No	Training	Dialogue analysis
[62]	Text	EN	2025	GPT-3.5	None	ZS, INST	No	Supplement	Phenotype ontology
[7]	Text	EN	2025	GPT-3.5/4, Llama-2, Mistral	None	ZS, FS	No	Training	Radiology prediction
[63]	Text	ZH	2025	GPT-3.5, Claude	None	FS, RAG	Request	Training	Anesthesia QA
[64]	Tabular	-	2025	HALO	SFT	INST	Yes	Supplement	Patient outcome prediction
[65]	Text	ZH	2025	ChatGPT	None	FS, INST	Yes	Training	Mental health IE
[66]	Tabular	EN	2025	GPT-4o	None	ZS, INST	Yes	Privacy	Data scarcity
[67]	Text	EN	2025	GPT-3.5/4, Mistral	None	FS, INST	No	Privacy	Phenotype inference
[68]	Text	EN	2025	GPT-4	None	CoT	Request*	Training	Medical reasoning
[69]	Text	ZH	2025	GPT-3.5	None	INST	Request	Training	Database search
[70]	Text	EN	2025	Llama3-instruct	None	FS	No	Training	Metastases detection
[71]	Image	EN	2025	Stable Diffusion	None	FS	Yes	Supplement	Colonoscopy analysis
[72]	Image	EN	2025	ChatGPT API	None	INST	No	Supplement	Pathology analysis
[73]	Text	ET	2025	GPT-2	TAFT	ZS, FS	Request	Training	Clinical IE
[74]	Tabular	EN	2025	DistilGPT-2	SFT	INST	No	Training	Patient outcome prediction

*Notes.* A dash (–) indicates unknown information. The “Request*” indicates data were stated to be publicly available but are either not yet released or temporarily inaccessible. Model names are reported as in the original papers; when only “ChatGPT” or “ChatGPT API” was mentioned, we list it accordingly

### Synthetic Data Types and Medical Applications

Increasing model developments for biomedical applications lead to the surging needs of synthetic data [26, 28]. In this section, we provide an overview of recent studies using synthetic data and address three critical questions: 1) What types of synthetic data are being generated? 2) What modalities and clinical tasks do these datasets target? 3) Are those data resources accessible for reusable research, such as benchmarking and model training? To answer those questions, we examine several aspects of the collected studies and summarize them in Table 1, including data modality, data language, data size, published year, medical application, and data or model accessibility. We aim to provide insights into current and future trends of data types and biomedical applications.

Biomedical synthetic data has shown diverse medical topics, languages, and modalities. Most of the studies (55.9%, $n=33$) are for general topics, while others include specific medical areas, such as suicide [11, 34], colorectal cancer [71], and radiology [7]. For example, studies generate synthetic data for augmenting diagnosis accuracy for Post-traumatic stress disorder (PTSD) [6] and Autism Spectrum Disorders (ASD) [60]. English synthetic data accounted for the majority of generated datasets (84.7%, $n=50$). Synthetic data were also generated in French [32], Chinese [37, 63, 65, 69], German [41], and Arabic [58], demonstrating initial efforts to support non-English language resources. For example, Moser et al. [41] employed multilingual LLMs to generate German emergency-medical dialogues simulating realistic interactions between ambulance staff and patients. Most datasets focus on unstructured text modality (e.g., clinical narratives [16, 29, 57] and discharge summaries [36]), while a smaller but growing subset includes tabular [35, 46, 48, 55, 59, 64, 66, 74], image [37, 71, 72], and audio [61]. For example, Theodorou et al. [35] generate novel tabular datasets of high-dimensional longitudinal EHR records to provide realistic, privacy-preserving alternatives for machine learning and statistical analysis, while Ejiga et al. [71] generate synthetic colonoscopy image datasets via fine-tuned text-to-image synthesis to augment training data for robust colorectal cancer detection and precise polyp segmentation. Data sizes vary across health issues (e.g., cancer and mental health), yet the majority has relatively small sets with fewer than 10K samples, indicating the critical utility of synthetic data under low-resourced scenarios. For example, FairPlay [64] generates synthetic data from authentic EHR records to bolster under-represented patient subgroups, boosting mortality-prediction F1 scores by up to 21% and markedly narrowing performance gaps across different demographic groups. The various synthetic datasets highlight a growing community to deploy the biomedical synthetic data.

Synthetic data generation in biomedical research increasingly supports a wide array of clinical applications. Those tasks cover diverse medical needs, including phenotype classification [15, 33, 52, 60, 70], PTSD symptom extraction [6], de-identification (SynNote) [28], clinical note summarization [36, 53], and mental health assessment [11, 12, 42]. For example, CALLM [6] constructs clinical interview datasets to aid PTSD diagnosis by generating synthetic transcripts; and Barabadi et al. [70] generated synthetic radiology reports to enable automatic detection of metastatic sites in cancer patients. Those applications broaden the utility of synthetic data for health challenges, such as data shortage [6] and privacy concerns [5, 13, 28, 66, 67]. However, accessibility remains a central concern for advancing synthetic data utility (see Supplementary Table S1 for a detailed list of experimental datasets and their corresponding links). Table 1 shows 50.8% ($n=30$) of the studies are explicitly described as accessible, providing open or partially open resources for reproducible development, while others’ accessibility details or licensing remain unclear. Open datasets such as Syn-HPI [27], ClinGen [14], and Syn-EMS-Audio [50] serve as synthetic data benchmarks for evaluating new models and methods, while others—particularly those derived from sensitive clinical domains—may be restricted or anonymized to protect patient privacy. Ensuring clear, well-documented access protocols and licenses will be essential for fostering broader collaboration and translational impacts of synthetic data in biomedical research.

### Synthetic Data Generation Methodology

**Fig. 2:**
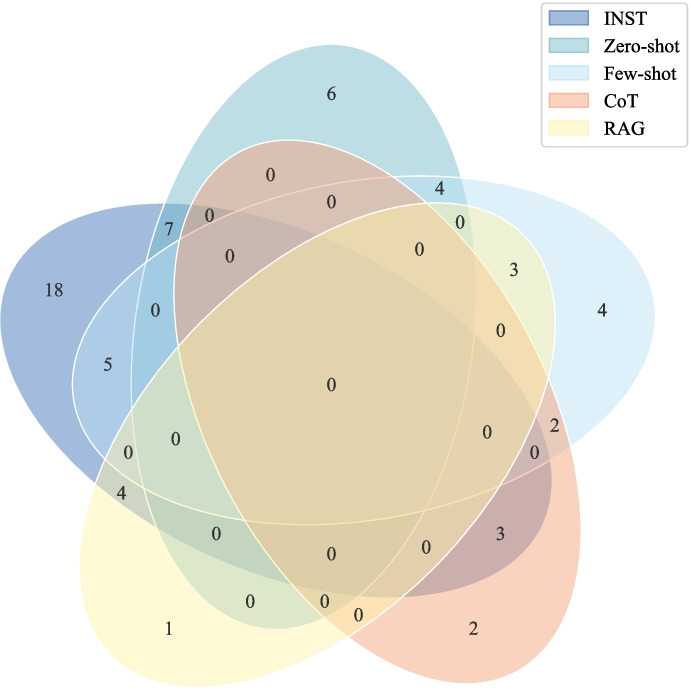
Overlap among prompting strategies (INST, Zero-shot, Few-shot, CoT, RAG) in the reviewed studies. Each intersection represents studies combining multiple methods

Emerging Transformer-based large language models (LLMs) have significantly advanced synthetic data generation methodologies in biomedical research, enabling diverse applications, as summarized in Table 1. These novel approaches address challenges associated with limited clinical data by augmenting datasets, thus enhancing generalizability and mitigating overfitting in downstream machine learning tasks across various healthcare domains. In this section, we systematically examine generation methodologies among 59 reviewed studies, summarizing recent trends in model architectures, generation techniques, and integration strategies for synthetic data generation. Specifically, we aim to answer two key questions: 1) what predominant methods (e.g., prompt-based vs. specialized architectures) are currently employed for biomedical synthetic data generation; and 2) what trends and variations do exist regarding model selection (open-source vs. closed-source) and prompting strategies (zero-shot, few-shot, reasoning-augmented)? Because several studies employed combinations of approaches (e.g., both open- and closed-source models, or few-shot with CoT or RAG), each study was counted in all applicable categories. In the case of prompting-based methods, studies employing multiple prompting strategies were summarized based on their two predominant prompting types for alignment, and the overlaps among these categories are visualized in Fig. 2.

Our review identifies prompt-based generation as the predominant synthetic data generation method, employed by approximately 74.6% ($n=44$) of the analyzed studies. Prompt-based approaches primarily rely on meticulously crafted textual instructions, leveraging fine-tuned LLMs, such as T5 and GPT variants (e.g., GPT-4) for diverse biomedical tasks [38, 59, 69]. The most prevalent strategy is instruction-driven (INST) prompting, observed in 42.4% ($n=25$) of studies, which guides generation through detailed, explicit instructions that typically encompass core task definitions, persona assignments, and specific constraints on the desired content, format, and style. It leverages the model’s ability to follow complex commands without in-context examples, making it highly versatile. For instance, Sufi et al.[36] utilized detailed instructions to generate realistic discharge summaries, while Wang et al.[56] employed structured prompts to synthesize patient portal messages for creating a corpus to analyze linguistic features and support downstream applications like patient portal message triage. Zero-shot prompting represents another approach, used in 28.8% ($n=17$) of studies, involving prompting LLMs without illustrative examples and relying solely on explicit task instructions and the pretrained knowledge encoded in LLMs [66]. This method is suitable for simpler tasks, such as dialogue summarization [29] or generating structured medical reports [57], where extensive contextual training is less critical. For example, Barr et al. [66] used zero-shot prompting with GPT-4o to synthesize perioperative clinical tables of patient demographics, surgical parameters and outcomes, demonstrating that the model can produce realistic synthetic data and preserve key statistical patterns based solely on qualitative instructions. Few-shot prompting provides a small number of curated examples (typically 2-5) within the prompt to illustrate the task and guide LLMs for synthetic data generation, which accounts for 20.3% ($n=12$) of studies. This approach uses limited curated examples to guide LLMs in generating clinically relevant synthetic data across diverse applications, including suicide symptom extractions [11], phenotype classification [15, 33, 52, 60, 70], and therapeutic dialogue generation [16, 42]. For example, Ghanadian et al.[11] use a patient’s suicidal narrative and a medication counseling dialogue as few-shot prompts to generate clinical texts for suicide symptom extraction and therapeutic dialogue generation, while Nievas et al.[39] use clinical trial inclusion and exclusion criteria paired with patient summaries as few-shot prompts to generate a synthetic patient–trial matching dataset. Beyond providing instructions and examples, more sophisticated techniques aim to enhance the reasoning and factuality of generated data. Reasoning-augmented prompting, commonly known as Chain-of-Thought (CoT) and used in 10.2% ($n=6$) of studies, enhances generation quality by instructing the model to first articulate its step-by-step reasoning process before producing the final output. This method improves the coherence and logical validity of synthetic data for complex clinical tasks. For example, Wu et al. [6] applied CoT to generate synthetic PTSD diagnostic interviews by prompting the model to provide both a simulated patient’s response and the clinical reasoning for it, ensuring the dialogue aligned with DSM-V criteria, while Kim et al.[68] used CoT to improve medical reasoning for question-answering tasks. Furthermore, retrieval-augmented generation (RAG), improves factual accuracy by dynamically incorporating information from external knowledge bases into the prompt. This technique, found in 11.9% ($n=7$) of studies, retrieves relevant documents (e.g., from clinical guidelines or medical literature) to ground the generation in verifiable, up-to-date information. For instance, Wang et al.[40] employed a RAG-like method for data augmentation, retrieving semantically similar synonyms from a knowledge graph to replace key terms. Similarly, Zafar et al.[44] enhanced medical QA by grounding generated answers with knowledge dynamically retrieved from the UMLS.

A notable trend is the model selection between open-source and closed-source LLMs, such as Llama vs GPT-4. Approximately 67.8% ($n=40$) of the reviewed studies directly utilize closed-source models such as GPT-3.5 and GPT-4 through proprietary APIs. This approach prioritizes rapid development and output quality without extensive model customization. The remaining 32.2% ($n=19$) of studies employ fine-tuning approaches on open-source models, which involves additional training of pretrained models on domain-specific datasets to adapt them for particular biomedical contexts. Fine-tuning approaches can be categorized into several distinct types based on their adaptation strategies. Supervised fine-tuning (SFT) involves training models on labeled, task-specific datasets where input-output pairs are explicitly provided [26, 64, 74]. Ive et al. [26] demonstrated this approach by fine-tuning Transformer models on real EHRs using a gap-filling task, where models learned to generate complete clinical documents conditioned on key phrases and clinical metadata for phenotype classification tasks. Domain-adaptive fine-tuning (DAFT) adapts models to broad clinical domains by training on large corpora of domain-specific text without task-specific labels [28, 32]. This approach helps models acquire clinical vocabulary, medical terminology, and domain-specific linguistic patterns. Libbi et al. [28] employed DAFT by fine-tuning an English GPT-2 model on Dutch clinical narratives with explicit PHI annotations, enabling joint generation of synthetic text and de-identification labels for training downstream NER models. Task-adaptive fine-tuning (TAFT) focuses on specific downstream tasks by training models on task-relevant data [25, 27, 30, 33, 35, 44, 73]. Lu et al. [30] applied TAFT to GPT-2 by prepending class labels to MIMIC-III discharge summaries during training, enabling the model to generate synthetic clinical notes with embedded readmission status for downstream prediction tasks. Instruction tuning (IT) trains models to follow structured commands and detailed instructions, enhancing their ability to generate content according to specific requirements [34, 43, 51, 57]. For instance, Jeong et al. [43] employed IT to fine-tune LLaMA2 models on medical instruction datasets, developing critic models that annotate and filter synthetic medical instructions to generate instruction sets for training generator models. Fine-tuned open-source models demonstrate particular advantages in privacy-sensitive clinical contexts and specialized medical domains. However, these models typically require more computational resources and domain expertise compared to direct API usage of closed-source models. Studies comparing model performance indicate that while fine-tuned open-source models offer greater customization potential, they generally underperform closed-source models on standardized clinical reasoning benchmarks. For example, Jeong et al. [43] observed that fine-tuned LLaMA2 7B models achieved lower performance than GPT-4 on clinical reasoning tasks, despite domain-specific adaptation.

Approximately 25.4% ($n=15$) of the reviewed studies fine-tuned pre-trained LLMs or explore specialized model architectures for synthetic data generation, including multimodal, multi-agent frameworks and custom-designed transformer architectures (e.g., HALO) [16, 26, 35, 48, 50, 61, 64, 71, 72]. Multimodal approaches integrate multiple data modalities, such as text, images, and audio, to create richer, more realistic synthetic datasets, such as colonoscopy image analysis [71], digital pathology interpretation [72], and emergency medical services (EMS) assistance [50]. For example, CognitiveEMS [50] prompts an LLM with emergency protocols to generate synthetic cardiac-arrest dialogues, converts them to speech, and synchronizes the audio with AR smart-glasses video frames annotated by a zero-shot vision classifier, producing a unified dataset for cardiac emergency analysis. Multi-agent frameworks [6, 16, 58] involve employing multiple interacting models or agents, each performing distinct roles, to collaboratively produce complex and realistic data, such as doctor-patient dialogues or clinical scenarios. For instance, NoteChat [16] employs a cooperative multi-agent framework with planning, role play, and refinement modules to generate synthetic patient–physician dialogues that mirror real clinical encounters. Custom transformer architectures[26, 35, 64] extend the standard decoder by integrating domain-specific metadata and employing hierarchical modeling strategies, first capturing broader structural information and then refining finer details, to generate structured and realistic synthetic clinical datasets tailored to specific medical scenarios. For example, Theodorou et al. [35] demonstrate that their hierarchical autoregressive model generates privacy-preserving EHRs whose temporal and code distributions yield downstream prediction performance on par with real data. Collectively, these specialized frameworks enhance the fidelity and applicability of synthetic biomedical data for various complex health scenarios.

**Table 2:** Evaluation characteristics of reviewed studies: disease, downstream task, data size, number of automated metrics (Metrics #), inclusion of intrinsic evaluation (Intrinsic+), human involvement in generation or task evaluations (Human-in-the-Loop), and LLM-based evaluation (LLM Eval). CLS, NER, IE, RE, QA, NLI, and ASR refer to downstream tasks of classification, named entity recognition, information extraction, relation extraction, question answering, natural language inference, and automatic speech recognition, respectively

Study	Disease	Task	Data Size (K)	Metrics #	Intrinsic+	Human-in-the-Loop	LLM Eval
[25]	General	QA	500,000	3	Yes	No	No
[26]	Mental health	CLS	11,000	6	Yes	Yes	No
[27]	General	NER	500	4	No	Yes	No
[28]	General	NER	355,000	5	No	Yes	No
[29]	General	Summarization	44800	3	No	Yes	No
[30]	General	CLS	48,393	3	No	No	No
[31]	General	RE, QA	20B	4	No	Yes	No
[32]	General	NER	4,946	6	Yes	Yes	No
[33]	Febrile convulsions	CLS	4,917	3	No	Yes	No
[34]	Suicide	CLS	50	2	No	Yes	No
[35]	General	CLS	975,788	8	No	Yes	No
[15]	Alzheimer	CLS	32,116	4	No	Yes	No
[36]	General	CLS	70	3	No	Yes	No
[6]	PTSD	CLS	1,900	3	No	Yes	No
[37]	Fundus Fluorescein Angiography	QA	4,110,581	5	No	Yes	No
[38]	Acute Renal Failure	CLS	9,000	7	No	Yes	No
[13]	General	CLS	4,033	5	No	No	No
[39]	General	CLS	2,000	8	No	Yes	No
[40]	General	NLI	-	5	No	No	No
[41]	General	-	200	1	Yes	No	No
[42]	Psychological Therapy	-	3,508	9	Yes	No	No
[43]	General	QA	18,854	3	No	No	No
[44]	General	QA	30,172	8	No	Yes	No
[14]	General	CLS, RE, NER	5,000	7	Yes	No	No
[45]	General	IE, CLS	1,800	3	No	Yes	No
[46]	COVID-19	CLS	2,050	1	No	Yes	No
[16]	General	Dialogue Generation	30,000	7	Yes	Yes	Yes
[47]	General	CLS	592	7	No	No	No
[48]	Gait-based Disease	Pose Estimation	14,400	4	Yes	No	No
[49]	General	IE	158,000	1	Yes	Yes	Yes
[50]	General	ASR	59-min	2	No	No	No
[51]	General	NER, RE	25,239	1	No	No	No
[11]	Suicide	CLS	4,897	2	No	Yes	No
[52]	Cancer	CLS	180	2	No	No	No
[53]	General	Summarization	5,256	4	Yes	Yes	Yes
[54]	General	QA	147,980	1	No	No	No
[55]	Skeleton pose	CLS	5,400	2	Yes	No	No
[56]	General	-	450	2	Yes	Yes	No
[57]	General	Summarization	100,000	6	No	No	Yes
[58]	General	QA	150	4	No	Yes	Yes
[59]	Breast cancer	CLS	-	6	Yes	No	No
[12]	Depression	Semantic Search	2,700	4	No	No	No
[60]	Autism	CLS	4,200	3	No	Yes	No
[5]	Stroke thrombolysis contraindications	CLS	150	6	No	Yes	No
[61]	General	CLS	3,042	4	No	Yes	No
[62]	Phenotype ontology	IE	458,574	3	No	No	No
[7]	Limb Fractures	CLS	4,600	3	Yes	No	Yes
[63]	Anesthesiology	QA	180,000	5	No	Yes	Yes
[64]	General	CLS	-	2	No	No	No
[65]	Mental health	IE	5,802	2	No	Yes	No
[66]	Perioperative clinical data	-	6,166	1	Yes	No	No
[67]	General	CLS	88,353	3	Yes	No	No
[68]	General	QA	77,776	5	No	Yes	Yes
[69]	General	CLS	30,000	2	No	No	No
[70]	Cancer	CLS	74,450	1	No	Yes	No
[71]	Colorectal cancer	CLS, Segmentation	1,800	7	No	No	No
[72]	Breast cancer	CLS, Segmentation	-	4	No	Yes	No
[73]	General	NER	4,100	3	No	Yes	No
[74]	Breast Cancer, Diabetes	CLS	1,165,660	3	No	No	No

### Quality Assessment and Evaluation Metrics

Ensuring the quality of the synthetic data is the key to building precise models and medical applications. In this section, we assess our collected studies from the synthetic data assessment and evaluation perspective, as shown in Table 2. We aim to answer the following three questions: 1) What metrics and evaluation approaches are commonly used for assessing synthetic data quality? 2) What are the major challenges in model evaluations using synthetic data across biomedical diseases and tasks; and 3) What are the emerging trends in synthetic data quality control for biomedical research? To answer those questions, we systematically examined the collected 59 studies by disease topics, types of evaluation metrics (automated and human approaches), downstream biomedical tasks, and emerging strategies (e.g., LLM-based and intrinsic evaluations). We aim to highlight current practices, identify persistent challenges, and discuss notable shifts toward more robust and clinically meaningful data and model assessments in the biomedical synthetic data generation.

We examined two major evaluation metrics across diverse biomedical applications, intrinsic and extrinsic metrics, which are the essential measurements to select synthetic data and computational models. Intrinsic metrics (e.g., perplexity [49] or similarity measures such as KL Divergence [7]) assess properties of the synthetic data independently from downstream tasks, while extrinsic metrics rely on downstream tasks, such as classification [26]. As shown in Table 2, we can observe that the intrinsic metrics are not widely adopted and only count 27.1% ($n=16$) of the studies. For example, Study [14] and Study [53] employ intrinsic metrics to directly compare the statistical distribution of real and synthetic datasets, while most works, such as Studies [30, 33], and [62], rely primarily on extrinsic evaluation through classification (CLS) or information extraction (IE) tasks. Commonly used metrics include accuracy [34], F1 score [33], BLEU [37], and other task-specific measurements, such as ROUGE [68] and AUCROC [30]. The studies cover heterogeneous downstream tasks, such as de-identification for privacy protection [13], phenotype prediction [62], disease entity extraction [49], and clinical note summarization [29, 53]. Privacy protection assessment was reported in 20.3% ($n=12$) of the studies, primarily through membership and attribute inference attacks [13], adversary test [35], and memorization evaluation [26]. Those studies cover multiple medical domains, with most studies focusing on general biomedical applications ($n=33$) and others targeting 25 specific medical conditions and health-related topics, such as Alzheimer’s [15], breast cancer [59], and depression [12].

**Fig. 3:**
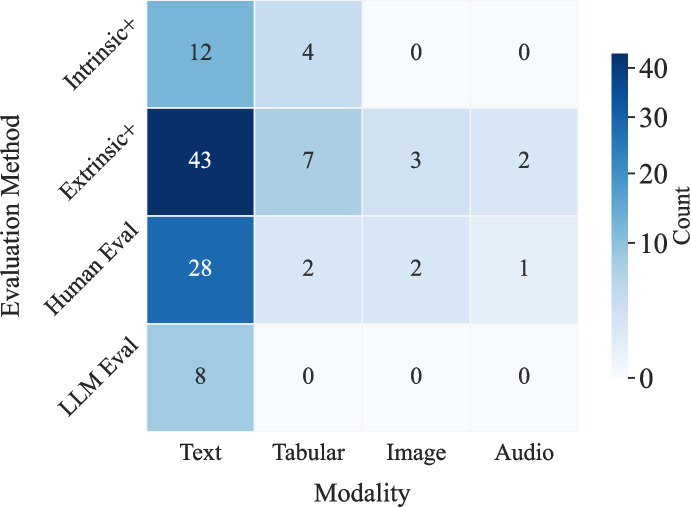
Heatmap linking data modality to evaluation method, summarizing the number of reviewed studies across four data modalities (Text, Tabular, Image, Audio) and four evaluation types: intrinsic evaluation (Intrinsic+), extrinsic evaluation (Extrinsic+), human evaluation (Human Eval; as in “Human-in-the-Loop” in Table 2), and LLM-based evaluation (LLM Eval)

Beyond automated metrics, human evaluation has played a selective yet important role in the assessment of synthetic biomedical data or its utility in different tasks (e.g., phenotype inference or clinical note summary), particularly for tasks where clinical relevance or nuanced interpretation is required. Human evaluation [15, 16, 39] typically involves domain experts to determine if synthetic data are meaningful, such as clinicians or biomedical researchers, who assess the synthetic data for factors like clinical validity. Human evaluation was included in a minority of studies (44.1%). Those studies cover tasks such as clinical entity recognition [27], clinical note summarization [29, 53], or question answering [44], where expert judgment adds valuable context beyond quantitative scores. More recently, a few studies [16, 49, 53, 57] (13.6% of our studies) explore using large language models (LLMs) as automated evaluators and leverage their ability to perform nuanced judgments, which approximates human assessment. Common LLM-based evaluation metrics employed in these studies include factual consistency [57], clinical correctness [49], and error identification [58], often measured through prompt-based scoring or rubric-guided assessments conducted by the LLM itself. For example, a recent study [57] prompts GPT-4 if the context supports / contradicts the response, or if there’s not enough information as a hallucination evaluation, whereas another study [49] uses a rubric-guided prompt, requesting GPT-4 to score each response on a four-level scale: Unacceptable, Poor, Satisfactory, and Excellent. However, LLM-based evaluations remain an emerging approach and have not yet replaced the need for statistical metrics or domain expert involvement, especially in clinically sensitive or complex scenarios [75]. The overall distribution of evaluation methods across data modalities is summarized in Fig. 3. As shown, text-based studies dominate all evaluation types, while non-text modalities such as tabular, image, and audio remain underrepresented, particularly in intrinsic and LLM-based evaluations.

Despite substantial progress, several challenges persist in the quality control of biomedical synthetic data. A key issue is the lack of standardized evaluation frameworks, resulting in wide variability in both the selection and reporting of metrics across studies and disease domains [14]. This heterogeneity complicates direct comparison between methods and limits the transferability of findings across medical applications and diseases. Additionally, Table 2 shows that studies still underutilize intrinsic evaluation, focusing instead on extrinsic, task-specific benchmarks that may not fully capture the utility or the limitations of synthetic data. Emerging trends suggest a gradual shift toward more comprehensive assessments, including extrinsic and intrinsic evaluation integrations, LLM-based metric adoption, and human professional engagements.

**Fig. 4:**
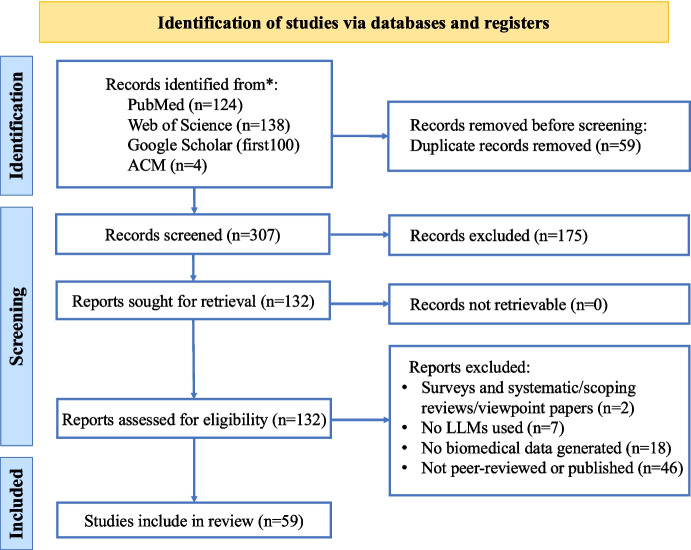
PRISMA-ScR flow diagram

## Methods

### Data Source and Search

We conduct searches between June and August 2024 with an additional search in May 2025 following the Preferred Reporting Items for Systematic Reviews and Meta-Analyses (PRISMA) guidelines [76] and the PRISMA Extension for Scoping Reviews (PRISMA-ScR) [77] (Fig. 4). Recognizing the rapid developments of Large Language Models (LLMs) in the biomedical field, our review encompassed peer-reviewed articles between January 1, 2020, and April 5, 2025 (inclusive) from multiple database sources, including ACM Digital Library, PubMed, Web of Science, and Google Scholar. Supplemental articles were gathered by reviewing article bibliographies and by soliciting suggestions from XH, HW, IH, and ZH. Our search strategy utilized a logical combination of keywords, and the full boolean search string used for Web of Science was: TS=(“large language model*” OR “LLM*”) AND TS=(synthetic*) AND TS=(data*) AND TS=(biomedical* OR medical* OR clinical* OR health*). Search strings for other databases followed a similar logic and are provided in the Appendix A (Table 3). We primarily limited the search to titles and abstracts, expanding to full texts where this function was unavailable. For Google Scholar, we conducted a keyword search, sorted by relevance, and selected the first 100 studies. The search strategies for each database were initially formulated in the early stages of the study and refined through team discussions and preliminary analysis of the results. The final database search was conducted on April 5, 2025, covering all publications available by that date.

### Study Selection

All records retrieved from the database searches were imported into Mendeley for reference management. Duplicates were removed in Mendeley based on matching titles, authors, and publication details before screening. Screening and eligibility assessment were conducted collaboratively using a shared Excel spreadsheet. Titles and abstracts were initially screened against predefined eligibility criteria by at least two independent reviewers, excluding records that clearly did not meet inclusion criteria and retaining ambiguous ones for full-text screening. Our inclusion criteria include: 1) an article conducts biomedical research; 2) an article uses Large Language Models (LLMs) for synthetic generation of biomedical data; and 3) an article has undergone peer review and been published in a journal or in conference proceedings within the range of 01/01/2020 and 04/05/2025. Our exclusion criteria include: 1) an article is a survey, literature review, news article, editorial, letter, opinion, study protocol, and comment; 2) an article is not in English; 3) articles do not study biomedical large language models; and 4) an article is not peer-reviewed and published in a journal or conference proceedings. Three undergraduate research assistants in the computer science major from the XH lab, who had significant experience and trainings in biomedical studies. They assisted HS and XH in screening candidate articles. Each record was independently reviewed by at least two individuals, with decisions documented in a shared spreadsheet. Discrepancies were discussed to reach consensus, and unresolved cases were adjudicated by XH or HW.

### Data Extraction

Selected articles were mainly processed by two individuals (HR and XH), who are experts in natural language processing (NLP) and biomedical informatics. They independently reviewed the full text of each selected article and extracted key metadata using a predefined data extraction template developed in Microsoft Excel. The template was pilot-tested on five randomly selected studies to ensure that all relevant information could be consistently captured and refined before full data extraction. The extracted data focused on three main aspects: 1) Synthetic data types and applications, including data characteristics , such as language and domain; 2) Synthetic data generation methods, presenting generation pipelines (models used to generate synthetic data) and their data modalities (e.g., text, image, audio); 3) Quality assessment and evaluation metrics summarizing medical applications, evaluation approaches, and evaluation settings. Each field in the template corresponded to one column in the summary tables (e.g., Tables 1 and 2), allowing for transparent and reproducible aggregation of results. Discrepancies in data extraction were resolved through discussion between HR and XH, with further arbitration by XH, HW, and IH when necessary. All extraction processes were documented for replication purposes.

## Discussion

Our scoping review has presented the current developments and applications of biomedical synthetic data generation, which is being facilitated by large language models (LLMs). In this section, we highlight several insightful takeaways of emerging methodological and evaluative trends and identify promising areas for future biomedical research and applications using synthetic data generation.

This review shows a clear shift towards leveraging synthetic data to overcome biomedical research challenges, such as data scarcity, privacy restrictions, and diverse clinical conditions. While synthetic data has been used effectively across varied clinical applications (e.g., phenotype ontology extraction [62] and cancer diagnosis [52, 71, 72]), the scale still varies across data types and disease fields, reflecting the nuanced capabilities and limitations of current LLMs. To navigate these complexities and enhance data quality, our review identifies a strategic adoption of advanced generation methodologies. Fine-tuning approaches, such as task-adaptive fine-tuning (TAFT), are employed to specialize models for specific clinical contexts, thereby improving domain relevance [25, 27, 30, 33]. In parallel, sophisticated prompting techniques address concerns of logical coherence and factuality. For instance, reasoning-augmented methods like Chain-of-Thought (CoT) improve the clinical plausibility of outputs [6, 68], while knowledge-augmented generation (RAG) grounds synthetic data in verifiable external sources to mitigate hallucination [40, 44]. These methods are applied to various data types, with clinical narratives and unstructured texts remaining the most common data source, due primarily to their widespread availability and the inherent strengths of LLMs in natural language processing tasks. We can also find an emerging trend toward multimodal and structured synthetic EHR, which indicates increasing expansions across more clinical settings, such as imaging, audio, and wearable sensors [48].

However, the advances indicate potential concerns in quality augmentation and assessments. Our review shows that current studies are significantly depending on prompting closed-source LLMs, like GPT-4. While few-shot and zero-shot approaches may achieve some successful cases [6, 38, 51], prompting closed-source LLMs (e.g., GPT-4) prioritizes rapid development and sophisticated output quality, though their proprietary nature poses challenges for transparency, reproducibility, and regulatory approval in clinical settings. This reliance on proprietary LLMs presents several challenges. Access to proprietary models may be restricted by cost, availability, or data governance policies. For example, MIMIC-IV (Medical Information Mart for Intensive Care) [78] does not allow for uploading data to those closed-source LLMs. Reproducibility faces additional barriers from the non-deterministic nature of these APIs and inconsistent reporting of prompts or decoding settings. Additionally, among the 43 studies that used prompt-based generation, many did not report essential details such as model versions, complete prompts used, or decoding parameters (e.g. sampling temperature), making replication difficult [36, 72]. Hallucination and fabrication risks often lack systematic evaluation, potentially leading to errors in downstream applications. Many studies rely on n-gram-based metrics such as ROUGE, which assess textual fluency but cannot verify clinical accuracy. More comprehensive factuality assessments, such as Hallucination Rate quantification or ontology-based checks like UMLS-F1 [53], were adopted by only a minority of studies, leaving a gap in ensuring the factual accuracy and clinical safety of synthetic biomedical data. Addressing these challenges requires implementing basic safeguards, such as disclosing generation details (e.g., prompts, model versions, decoding parameters), avoiding same-family generator–evaluator configurations, and conducting targeted expert reviews to assess factual accuracy. Future work should focus on developing and benchmarking open-source alternatives and exploring methods that reduce reliance on proprietary systems.

Our review indicates future research in biomedical synthetic data generation may evolve along three dimensions. There is a clear trend towards generating multimodal synthetic datasets to simulate more real-world and complex clinical scenarios, such as radiology [7, 70], pathology [72], and emergency care [41]. Future synthetic data generation may depend on more precise LLM-based systems by knowledge-guided generations [14], multi-agent frameworks [16], and human-LLM collaborations [46], which leverage clinical knowledge to enhance factuality and specificity of synthetic data qualities. In terms of evaluation, more standardized, multi-dimensional, and human-centered assessments are critical. Future studies on biomedical synthetic data generation may consider a mixture of intrinsic and extrinsic metrics, human-in-the-loop approaches, and task-agnostic validations to ensure fidelity, utility, and privacy of synthetic data. We envision the directions will be essential to advance biomedical research and applications by the synthetic data generations and LLMs.

### Limitations

This scoping review should be interpreted in light of several constraints. First, the search was restricted to four major databases (ACM Digital Library, PubMed, Web of Science, and Google Scholar) and to peer-reviewed venues; grey literature, non-indexed conference proceedings, and pre-prints may therefore be under-represented. Second, we limited inclusion to studies published in English between 1 January 2020 and 5 April 2025, so potentially relevant work in other languages or outside this time frame was not captured. Third, consistent with the PRISMA-ScR framework, we performed no critical appraisal of individual study quality; consequently, the methodological robustness of the included papers was not formally assessed. Additionally, the heterogeneity of study designs, synthetic-data tasks, evaluation metrics, and biomedical application domains precluded quantitative synthesis and limits the generalizability of pooled observations. Reproducibility is further limited by reliance on proprietary, non-deterministic APIs and incomplete reporting of prompts/decoding settings; hallucination audits were inconsistently performed across included studies. Lastly, given the rapid pace of innovation in LLM-based synthetic data generation, newly published methods appearing after our search window may not be reflected here, underscoring the need for periodic updates of this evidence map (Table 3).

## Conclusion

Our scoping review examines the current trends and challenges of utilizing synthetic data generated by language models in biomedical fields with a focus on utility and quality aspects. The results indicate that to fully utilize the LLM-generated synthetic data, the field may address several critical issues, including standardized evaluation frameworks, increased transparency, human-AI collaboration, and accessible models and data. Although our review was limited by the rapid pace of advancements and a primary focus on English-language publications, these challenges present important opportunities for collaboration and innovation. By promoting reproducibility, rigorous evaluation, and expert feedback for the synthetic generation approach, our study shows the integration of high-quality synthetic data can benefit biomedical research and clinical practice.

## Supplementary Information

Below is the link to the electronic supplementary material.

Supplementary file1 (XLSX 26 KB)

## Data Availability

No datasets were generated or analysed during the current study.

## Appendix A Database-Specific Search Strategies

**Table 3:** Search strategies for each database included in this review

Database	Search string
PubMed	(“large language model*”[Title/Abstract] OR “LLM*”[Title/Abstract]) AND (synthetic*[Title/Abstract]) AND (data*[Title/Abstract]) AND (biomedical*[Title/Abstract] OR medical*[Title/Abstract] OR clinical*[Title/Abstract] OR health*[Title/Abstract])
ACM Digital Library	Abstract:(“large language model*” OR “LLM*”) AND Abstract:synthetic* AND Abstract:data* AND Abstract:(biomedical* OR medical* OR clinical* OR health*)
Web of Science	TS=(“large language model*” OR “LLM*”) AND TS=(synthetic*) AND TS=(data*) AND TS=(biomedical* OR medical* OR clinical* OR health*)
Google Scholar	(“large language model” OR “large language models” OR LLM) AND synthetic AND data AND (biomedical OR medical OR clinical OR health)

## References

[1] Liu W, He Z, Huang X (2024) Time matters: Examine temporal effects on biomedical language models, pp. 723–732. American Medical Informatics Association, San Francisco, CA, USA (2024). http://www.ncbi.nlm.nih.gov/pubmed/40417490 PMC12099427 40417490

[2] Jones P, Liu W, Huang I-C, Huang X (2025) Examining imbalance effects on performance and demographic fairness of clinical language models. In: 2025 IEEE 13th International Conference on Healthcare Informatics (ICHI), pp 58–68. 10.1109/ICHI64645.2025.00016

[3] Stade EC, Stirman SW, Ungar LH, Boland CL, Schwartz HA, Yaden DB, Sedoc J, DeRubeis RJ, Willer R, Eichstaedt JC (2024) Large language models could change the future of behavioral healthcare: a proposal for responsible development and evaluation. npj Mental Health Res. 3(1):12. 10.1038/s44184-024-00056-z 10.1038/s44184-024-00056-z PMC10987499 38609507

[4] Blanco-Gonzalez A, Cabezon A, Seco-Gonzalez A, Conde-Torres D, Antelo-Riveiro P, Pineiro A, Garcia-Fandino R (2023) The role of ai in drug discovery: challenges, opportunities, and strategies. Pharmaceuticals 16(6):891. 10.3390/ph16060891 37375838 10.3390/ph16060891 PMC10302890

[5] Chen BY, Antaki F, Gonzalez M, Uchino K, Albahra S, Robertson S, Ibrikji S, Aube E, Russman A, Hussain MS (2025) Automated identification of stroke thrombolysis contraindications from synthetic clinical notes: A proof-of-concept study. Cerebrovasc Diseases Extra. 15:130–136. 10.1159/000545317 10.1159/000545317 PMC12021381 40096831

[6] Wu Y, Mao K, Zhang Y, Chen J (2024) Callm: Enhancing clinical interview analysis through data augmentation with large language models. IEEE J Biomed Health Inform. 10.1109/JBHI.2024.3435085 10.1109/JBHI.2024.3435085 39074002

[7] Liu J, Koopman B, Brown NJ, Chu K, Nguyen A (2025) Generating synthetic clinical text with local large language models to identify misdiagnosed limb fractures in radiology reports. Artif Intell Med. 159 10.1016/j.artmed.2024.103027 10.1016/j.artmed.2024.103027 39580897

[8] Achiam J, Adler S, Agarwal S, Ahmad L, Akkaya I, Aleman FL, Almeida D, Altenschmidt J, Altman S, et al (2023) GPT-4 Technical Report. Technical report, OpenAI. arXiv:2303.08774

[9] Dubey A, Grattafiori A, Jauhri A, Pandey A, Kadian A, Al-Dahle A, Touvron H, et al (2024) The llama 3 herd of models. Technical report, Meta AI. arXiv:2407.21783

[10] Han G, Liu W, Huang X, Borsari B (2024) Chain-of-interaction: Enhancing large language models for psychiatric behavior understanding by dyadic contexts. In: Proceedings of the 12th IEEE International Conference on Healthcare Informatics (ICHI), pp 392–401. IEEE, Orlando, FL, USA. 10.1109/ICHI61247.2024.00057

[11] Ghanadian H, Nejadgholi I, Osman HA (2024) Socially aware synthetic data generation for suicidal ideation detection using large language models. IEEE Access. 12:14350–14363. 10.1109/ACCESS.2024.3358206

[12] Bucur A-M (2024) Leveraging llm-generated data for detecting depression symptoms on social media. In: Experimental IR Meets Multilinguality, Multimodality, and Interaction: 15th International Conference of the CLEF Association, CLEF 2024, Grenoble, France, September 9–12, 2024, Proceedings, Part I, pp 193–204. Springer, Berlin, Heidelberg. 10.1007/978-3-031-71736-9_14

[13] Sarkar AR, Chuang Y-S, Mohammed N, Jiang X (2024) De-identification is not enough: a comparison between de-identified and synthetic clinical notes. Sci Rep 14(1):29669. 10.1038/s41598-024-81170-y 10.1038/s41598-024-81170-y PMC11607336 39613846

[14] Xu R, Cui H, Yu Y, Kan X, Shi W, Zhuang Y, Wang MD, Jin W, Ho J, Yang C (2024) Knowledge-infused prompting: Assessing and advancing clinical text data generation with large language models. In: Ku L-W, Martins A, Srikumar V (eds) Findings of the Association for Computational Linguistics: ACL 2024, pp 15496–15523. Association for Computational Linguistics, Bangkok, Thailand. 10.18653/v1/2024.findings-acl.916 10.18653/v1/2024.findings-acl.916 PMC12949634 41766793

[15] Li R, Wang X, Yu H (2023) Two directions for clinical data generation with large language models: Data-to-label and label-to-data. In: Bouamor H, Pino J, Bali K (eds) Findings of the Association for Computational Linguistics: EMNLP 2023, pp 7129–7143. Association for Computational Linguistics, Singapore. 10.18653/v1/2023.findings-emnlp.474 10.18653/v1/2023.findings-emnlp.474 PMC10782150 38213944

[16] Wang J, Yao Z, Yang Z, Zhou H, Li R, Wang X, Xu Y, Yu H (2024) NoteChat: A dataset of synthetic patient-physician conversations conditioned on clinical notes. In: Ku L-W, Martins A, Srikumar V (eds) Findings of the Association for Computational Linguistics: ACL 2024, pp 15183–15201. Association for Computational Linguistics, Bangkok, Thailand. 10.18653/v1/2024.findings-acl.901 10.18653/v1/2024.findings-acl.901 PMC13248957 42273713

[17] Figueira A, Vaz B (2022) Survey on synthetic data generation, evaluation methods and gans. Mathematics 10(15):2733. 10.3390/math10152733

[18] Long L, Wang R, Xiao R, Zhao J, Ding X, Chen G, Wang H (2024) On LLMs-driven synthetic data generation, curation, and evaluation: A survey. In: Ku L-W, Martins A, Srikumar V (eds) Findings of the Association for Computational Linguistics: ACL 2024, pp 11065–11082. Association for Computational Linguistics, Bangkok, Thailand. 10.18653/v1/2024.findings-acl.658

[19] Li Z, Zhu H, Lu Z, Yin M (2023) Synthetic data generation with large language models for text classification: Potential and limitations. In: Bouamor H, Pino J, Bali K (eds) Proceedings of the 2023 Conference on Empirical Methods in Natural Language Processing, pp 10443–10461. Association for Computational Linguistics, Singapore. 10.18653/v1/2023.emnlp-main.647

[20] Murtaza H, Ahmed M, Khan NF, Murtaza G, Zafar S, Bano A (2023) Synthetic data generation: State of the art in health care domain. Comput Sci Rev. 48:100546 10.1016/j.cosrev.2023.100546

[21] Gonzales A, Guruswamy G, Smith SR (2023) Synthetic data in health care: A narrative review. PLOS Digital Health 2(1):1–16. 10.1371/journal.pdig.0000082 10.1371/journal.pdig.0000082 PMC9931305 36812604

[22] Smolyak D, Bjarnadóttir MV, Crowley K, Agarwal R (2024) Large language models and synthetic health data: progress and prospects. JAMIA Open 7(4):114. 10.1093/jamiaopen/ooae114 10.1093/jamiaopen/ooae114 PMC11512648 39464796

[23] Loni M, Poursalim F, Asadi M, Gharehbaghi A (2025) A review on generative AI models for synthetic medical text, time series, and longitudinal data. npj Digital Med. 8(1):281 10.1038/s41746-024-01409-w 10.1038/s41746-024-01409-w PMC12081667 40374917

[24] Landis JR, Koch GG (1977) The measurement of observer agreement for categorical data. Biometrics 33(1):159–174. 10.2307/2529310 843571

[25] Shakeri S, Santos C, Zhu H, Ng P, Nan F, Wang Z, Nallapati R, Xiang B (2020) End-to-end synthetic data generation for domain adaptation of question answering systems. In: Webber B, Cohn T, He Y, Liu Y (eds) Proceedings of the 2020 Conference on Empirical Methods in Natural Language Processing (EMNLP), pp 5445–5460. Association for Computational Linguistics, Online. 10.18653/v1/2020.emnlp-main.439

[26] Ive J, Viani N, Kam J, Yin L, Verma S, Puntis S, Cardinal RN, Roberts A, Stewart R, Velupillai S (2020) Generation and evaluation of artificial mental health records for natural language processing. npj Digit Med. 3 10.1038/s41746-020-0267-x 10.1038/s41746-020-0267-x PMC7224173 32435697

[27] Li J, Zhou Y, Jiang X, Natarajan K, Pakhomov SV, Liu H, Xu H (2021) Are synthetic clinical notes useful for real natural language processing tasks: A case study on clinical entity recognition. J Am Med Inf Ass. 28:2193–2201. 10.1093/jamia/ocab112 10.1093/jamia/ocab112 PMC8449609 34272955

[28] Libbi CA, Trienes J, Trieschnigg D, Seifert C (2021) Generating synthetic training data for supervised de-identification of electronic health records. Future Int 13 10.3390/fi13050136

[29] Chintagunta B, Katariya N, Amatriain X, Kannan A (2021) Medically aware GPT-3 as a data generator for medical dialogue summarization. In: Shivade C, Gangadharaiah R, Gella S, Konam S, Yuan S, Zhang Y, Bhatia P, Wallace B (eds) Proceedings of the Second Workshop on Natural Language Processing for Medical Conversations, pp 66–76. Association for Computational Linguistics, Online. 10.18653/v1/2021.nlpmc-1.9

[30] Lu Q, Dou D, Nguyen TH (2021) Textual data augmentation for patient outcomes prediction. In: 2021 IEEE International Conference on Bioinformatics and Biomedicine (BIBM), pp 2817–2821. 10.1109/BIBM52615.2021.9669861

[31] Peng C, Yang X, Chen A, Smith KE, PourNejatian N, Costa AB, Martin C, Flores MG, Zhang Y, Magoc T, Lipori G, Mitchell DA, Ospina NS, Ahmed MM, Hogan WR, Shenkman EA, Guo Y, Bian J, Wu Y (2023) A study of generative large language model for medical research and healthcare. npj Digital Med. 6(1):210 10.1038/s41746-023-00958-w 10.1038/s41746-023-00958-w PMC10654385 37973919

[32] Hiebel N, Ferret O, Fort K, Névéol A (2023) Can synthetic text help clinical named entity recognition? a study of electronic health records in French. In: Vlachos A, Augenstein I (eds) Proceedings of the 17th Conference of the European Chapter of the Association for Computational Linguistics, pp 2320–2338. Association for Computational Linguistics, Dubrovnik, Croatia . 10.18653/v1/2023.eacl-main.170

[33] Khademi S, Palmer C, Dimaguila GL, Javed M, Buttery J, Black J (2023) Data augmentation to improve syndromic detection from emergency department notes. In: Proceedings of the 2023 Australasian Computer Science Week. ACSW ’23, pp 198–205. Association for Computing Machinery, New York, NY, USA. 10.1145/3579375.3579401

[34] Spitale G, Schneider G, Germani F, Biller-Andorno N (2023) Exploring the role of ai in classifying, analyzing, and generating case reports on assisted suicide cases: feasibility and ethical implications. Frontier Artif Intell 6 10.3389/frai.2023.1328865 10.3389/frai.2023.1328865 PMC10757918 38164497

[35] Theodorou B, Xiao C, Sun J (2023) Synthesize high-dimensional longitudinal electronic health records via hierarchical autoregressive language model. Nature Commun 14(1). 10.1038/s41467-023-41093-0 10.1038/s41467-023-41093-0 PMC10471716 37652934

[36] Sufi F (2024) Addressing data scarcity in the medical domain: A gpt-based approach for synthetic data generation and feature extraction. Information (Switzerland). 15 10.3390/info15050264

[37] Chen X, Xu P, Li Y, Zhang W, Song F, He M, Shi D (2024) Chatffa: An ophthalmic chat system for unified vision-language understanding and question answering for fundus fluorescein angiography. iScience. 27(7):110021 10.1016/j.isci.2024.110021 10.1016/j.isci.2024.110021 PMC11269310 39055931

[38] Litake O, Park BH, Tully JL, Gabriel RA (2024) Constructing synthetic datasets with generative artificial intelligence to train large language models to classify acute renal failure from clinical notes. J Am Med Inform Assoc 31(6):1404–1410. 10.1093/jamia/ocae081 38622901 10.1093/jamia/ocae081 PMC11105122

[39] Nievas M, Basu A, Wang Y, Singh H (2024) Distilling large language models for matching patients to clinical trials. J Am Med Inform Assoc 31(9):1953–1963. 10.1093/jamia/ocae073 38641416 10.1093/jamia/ocae073 PMC11339497

[40] Wang Y, Wang Z, Wang W, Chen Q, Huang K, Nguyen A, De S (2024) DKE-research at SemEval-2024 task 2: Incorporating data augmentation with generative models and biomedical knowledge to enhance inference robustness. In: Ojha AK, Doğruöz AS, Tayyar Madabushi H, Da San Martino G, Rosenthal S, Rosá A (eds) Proceedings of the 18th International Workshop on Semantic Evaluation (SemEval-2024), pp 88–94. Association for Computational Linguistics, Mexico City, Mexico. 10.18653/v1/2024.semeval-1.15

[41] Moser D, Bender M, Sariyar M (2024) Generating synthetic healthcare dialogues in emergency medicine using large language models. Stud Health Technol Inf 321:235–239. 10.3233/SHTI241099 10.3233/SHTI241099 39575815

[42] Bird JJ, Wright D, Sumich A, Lotfi A (2024) Generative ai in psychological therapy: Perspectives on computational linguistics and large language models in written behaviour monitoring. In: Proceedings of the 17th International Conference on PErvasive Technologies Related to Assistive Environments. PETRA ’24, pp 322–328. Association for Computing Machinery, New York, NY, USA. 10.1145/3652037.3663893

[43] Jeong M, Sohn J, Sung M, Kang J (2024) Improving medical reasoning through retrieval and self-reflection with retrieval-augmented large language models. Bioinformatics. 40:119–129 10.1093/bioinformatics/btae238 10.1093/bioinformatics/btae238 PMC11211826 38940167

[44] Zafar A, Sahoo SK, Bhardawaj H, Das A, Ekbal A (2024) Ki-mag: A knowledge-infused abstractive question answering system in medical domain. Neurocomputing. 571 10.1016/j.neucom.2023.127141

[45] Guevara M, Chen S, Thomas S, Chaunzwa TL, Franco I, Kann BH, Moningi S, Qian JM, Goldstein M, Harper S et al (2024) Large language models to identify social determinants of health in electronic health records. NPJ Digital Med 7(1):6. 10.1038/s41746-023-00970-0 10.1038/s41746-023-00970-0 PMC10781957 38200151

[46] Ehrett C, Hegde S, Andre K, Liu D, Wilson T (2024) Leveraging open-source large language models for data augmentation in hospital staff surveys: Mixed methods study. JMIR Med Educ. 10:51433–51433 10.2196/51433 10.2196/51433 PMC11590755 39560937

[47] Gabriel RA, Litake O, Simpson S, Burton BN, Waterman RS, Macias AA (2024) On the development and validation of large language model-based classifiers for identifying social determinants of health. Proceed National Acad Sci United States of America. 121 10.1073/pnas.2320716121 10.1073/pnas.2320716121 PMC11441499 39284061

[48] Gao Y, Zhang W, Ren J, Zheng R, Jin Y, Wu D, Shu L, Xu X, Jin Z (2024) Pressinpose: Integrating pressure and inertial sensors for full-body pose estimation in activities. Proc. ACM Interact Mob Wearable Ubiquit Technol 8(4) 10.1145/3699773

[49] Kweon S, Kim J, Kim J, Im S, Cho E, Bae S, Oh J, Lee G, Moon JH, You SC, Baek S, Han CH, Jung YB, Jo Y, Choi E (2024) Publicly shareable clinical large language model built on synthetic clinical notes. In: Ku L-W, Martins A, Srikumar V (eds) Findings of the Association for Computational Linguistics: ACL 2024, pp 5148–5168. Association for Computational Linguistics, Bangkok, Thailand. 10.18653/v1/2024.findings-acl.305

[50] Weerasinghe K, Janapati S, Ge X, Kim S, Iyer S, Stankovic JA, Alemzadeh H (2024) Real-time multimodal cognitive assistant for emergency medical services. In: 2024 IEEE/ACM Ninth International Conference on Internet-of-Things Design and Implementation (IoTDI), pp 85–96. 10.1109/IoTDI61053.2024.00012

[51] Delmas M, Wysocka M, Freitas A (2024) Relation extraction in underexplored biomedical domains: A diversity-optimized sampling and synthetic data generation approach. Comput Linguist 50(3):953–1000. 10.1162/coli_a_00520

[52] Zeinali N, Albashayreh A, Fan W, White SG (2024) Symptom-bert: Enhancing cancer symptom detection in ehr clinical notes. J Pain Symp Manage 68:190–1981 10.1016/j.jpainsymman.2024.05.015 10.1016/j.jpainsymman.2024.05.015 PMC12433187 38789092

[53] Mishra P, Yao Z, Vashisht P, Ouyang F, Wang B, Mody VD, Yu H (2024) SYNFAC-EDIT: Synthetic imitation edit feedback for factual alignment in clinical summarization. In: Al-Onaizan Y, Bansal M, Chen Y-N (eds) Proceedings of the 2024 Conference on Empirical Methods in Natural Language Processing, pp 20061–20083. Association for Computational Linguistics, Miami, Florida, USA. 10.18653/v1/2024.emnlp-main.1120 10.18653/v1/2024.emnlp-main.1120 PMC12854549 41624007

[54] Zhang J, Cui W, Huang Y, Das K, Kumar S (2024) Synthetic knowledge ingestion: Towards knowledge refinement and injection for enhancing large language models. In: Al-Onaizan Y, Bansal M, Chen Y-N (eds) Proceedings of the 2024 Conference on Empirical Methods in Natural Language Processing, pp 21456–21473. Association for Computational Linguistics, Miami, Florida, USA. 10.18653/v1/2024.emnlp-main.1196

[55] Dobhal U, Garcia C, Inoue S (2024) Synthetic skeleton data generation using large language model for nurse activity recognition. In: Companion of the 2024 on ACM International Joint Conference on Pervasive and Ubiquitous Computing. UbiComp ’24, pp 493–499. Association for Computing Machinery, New York, NY, USA. 10.1145/3675094.3678445

[56] Wang N, Treewaree S, Zirikly A, Lu YL, Nguyen MH, Agarwal B, Shah J, Stevenson JM, Taylor CO (2024) Taxonomy-based prompt engineering to generate synthetic drug-related patient portal messages. J Biomed Inf. 160 10.1016/j.jbi.2024.104752 10.1016/j.jbi.2024.104752 39603549

[57] Jones E, Palangi H, Simões C, Chandrasekaran V, Mukherjee S, Mitra A, Awadallah A, Kamar E (2024) Teaching language models to hallucinate less with synthetic tasks. In: 12th International Conference on Learning Representations, ICLR 2024, p 12. https://openreview.net/forum?id=xpw7V0P136

[58] Alghamdi HM, Mostafa A (2024) Towards reliable healthcare llm agents: A case study for pilgrims during hajj. Information (Switzerland). 15 10.3390/info15070371

[59] Wang Y, Fu T, Xu Y, Ma Z, Xu H, Du B, Lu Y, Gao H, Wu J, Chen J (2024) Twin-gpt: Digital twins for clinical trials via large language model. ACM Trans Multimed Comput Commun Appl. 10.1145/3674838

[60] Woolsey CR, Bisht P, Rothman J, Leroy G (2024) Utilizing Large Language Models to Generate Synthetic Data to Increase the Performance of BERT-Based Neural Networks. AMIA Joint Summits on Translational Science proceedings. AMIA Joint Summits on Transl Sci. 2024, 429–438 PMC11141799 38827067

[61] Scroggins JK, Topaz M, Song J, Zolnoori M (2024) Does synthetic data augmentation improve the performances of machine learning classifiers for identifying health problems in patient-nurse verbal communications in home healthcare settings? J Nurs Scholarsh 57(1):47–58. 10.1111/jnu.13004 38961517 10.1111/jnu.13004 PMC12849707

[62] Albayrak A, Xiao Y, Mukherjee P, Barnett SS, Marcou CA, Hart SN (2025) Enhancing human phenotype ontology term extraction through synthetic case reports and embedding-based retrieval: A novel approach for improved biomedical data annotation. J Pathol Inf. 16 10.1016/j.jpi.2024.100409 10.1016/j.jpi.2024.100409 PMC11667693 39720417

[63] Wang Z, Jiang J, Zhan Y, Zhou B, Li Y, Zhang C, Yu B, Ding L, Jin H, Peng J, Lin X, Liu W (2025) Hypnos: A domain-specific large language model for anesthesiology. Neurocomputing. 624 10.1016/j.neucom.2025.129389

[64] Theodorou B, Danek B, Tummala V, Kumar SP, Malin B, Sun J (2025) Improving medical machine learning models with generative balancing for equity and excellence. npj Digital Med. 8 (2025) 10.1038/s41746-025-01438-z 10.1038/s41746-025-01438-z PMC11828851 39953146

[65] Cai Z, Fang H, Liu J, Xu G, Long Y, Guan Y, Ke T (2025) Improving unified information extraction in chinese mental health domain with instruction-tuned llms and type-verification component. Artif Intell Med. 162 10.1016/j.artmed.2025.103087 10.1016/j.artmed.2025.103087 39987777

[66] Barr AA, Quan J, Guo E, Sezgin E (2025) Large language models generating synthetic clinical datasets: a feasibility and comparative analysis with real-world perioperative data. Frontier Artif Intell. 8 10.3389/frai.2025.1533508 10.3389/frai.2025.1533508 PMC11836953 39974356

[67] Chuang Y-S, Sarkar AR, Hsu Y-C, Mohammed N, Jiang X (2025) Robust privacy amidst innovation with large language models through a critical assessment of the risks. J Am Med Inform Assoc 32(5):885–892. 10.1093/jamia/ocaf037 40112189 10.1093/jamia/ocaf037 PMC12012348

[68] Kim H, Hwang H, Lee J, Park S, Kim D, Lee T, Yoon C, Sohn J, Park J, Reykhart O et al (2025) Small language models learn enhanced reasoning skills from medical textbooks. NPJ Digital Med 8(1):240. 10.1038/s41746-025-01653-8 10.1038/s41746-025-01653-8 PMC12048634 40316765

[69] Li J, Wan Z, Yu L, Liu H, Song H (2025) Synthetic data-driven approaches for chinese medical abstract sentence classification: Computational study. JMIR Format Res. 9 10.2196/54803 10.2196/54803 PMC11939029 40106267

[70] Barabadi MA, Zhu X, Chan WY, Simpson AL, Do RKG (2025) Targeted generative data augmentation for automatic metastases detection from free-text radiology reports. Frontier Artif Intell. 8 10.3389/frai.2025.1513674 10.3389/frai.2025.1513674 PMC11839598 39981192

[71] Peter OOE, Adeniran OT, John-Otumu AMG, Khalifa F, Rahman MM (2025) Text-guided synthesis in medical multimedia retrieval: A framework for enhanced colonoscopy image classification and segmentation. Algorithms. 18 10.3390/a18030155

[72] Li J, Zhu C, Zheng S, Chen P, Sun Y, Li H, Yang L (2025) Topofm: Topology-guided pathology foundation model for high-resolution pathology image synthesis with cellular-level control. IEEE Trans Med Imaging. 10.1109/TMI.2025.3548872 10.1109/TMI.2025.3548872 40048327

[73] Šuvalov H, Lepson M, Kukk V, Malk M, Ilves N, Kuulmets HA, Kolde R (2025) Using synthetic health care data to leverage large language models for named entity recognition: Development and validation study. J Med Int Res. 27 10.2196/66279 10.2196/66279 PMC11962312 40101227

[74] Miletic M, Sariyar M (2025) Utility-based analysis of statistical approaches and deep learning models for synthetic data generation with focus on correlation structures: Algorithm development and validation. JMIR AI. 4 10.2196/65729 10.2196/65729 PMC11969122 40112290

[75] Chen X, Xiang J, Lu S, Liu Y, He M, Shi D (2025) Evaluating large language models and agents in healthcare: key challenges in clinical applications. Intell Med 5(2):151–163. 10.1016/j.imed.2025.03.002

[76] ...Page MJ, McKenzie JE, Bossuyt PM, Boutron I, Hoffmann TC, Mulrow CD, Shamseer L, Tetzlaff JM, Akl EA, Brennan SE, Chou R, Glanville J, Grimshaw JM, Hrobjartsson A, Lalu MM, Li T, Loder EW, Mayo-Wilson E, McDonald S, McGuinness LA, Stewart LA, Thomas J, Tricco AC, Welch VA, Whiting P, Moher D (2021) The PRISMA 2020 statement: an updated guideline for reporting systematic reviews. BMJ 169(7):71. 10.1136/bmj.n71

[77] Tricco AC, Lillie E, Zarin W, O’Brien KK, Colquhoun H, Levac D, Moher D, Peters MD, Horsley T, Weeks L et al (2018) Prisma extension for scoping reviews (prisma-scr): Checklist and explanation. Ann Intern Med 169(7):467–473. 10.7326/M18-0850 30178033 10.7326/M18-0850

[78] Johnson AEW, Bulgarelli L, Shen L, Gayles A, Shammout A, Horng S, Pollard TJ, Hao S, Moody B, Gow B, Lehman L-wH, Celi LA, Mark RG (2023) MIMIC-IV, a freely accessible electronic health record dataset. Sci Data. 10(1):1 10.1038/s41597-022-01899-x 10.1038/s41597-022-01899-x PMC9810617 36596836

